# The Connection Between the Oral Microbiota and the Kynurenine Pathway: Insights into Oral and Certain Systemic Disorders

**DOI:** 10.3390/cimb46110750

**Published:** 2024-11-07

**Authors:** Rita Kis-György, Tamás Körtési, Alexandra Anicka, Gábor Nagy-Grócz

**Affiliations:** 1 Section of Health Behaviour and Health Promotion, Faculty of Health Sciences and Social Studies, University of Szeged, Temesvári krt. 31., H-6726 Szeged, Hungary; kis-gyorgy.rita@szte.hu; 2 Doctoral School of Interdisciplinary Medicine, University of Szeged, Szőkefalvi–Nagy Béla u. 4/B, H-6720 Szeged, Hungary; 3 Department of Theoretical Health Sciences and Health Management, Faculty of Health Sciences and Social Studies, University of Szeged, Temesvári krt. 31., H-6726 Szeged, Hungary; kortesi.tamas@szte.hu; 4 Preventive Health Sciences Research Group, Incubation Competence Centre of the Centre of Excellence for Interdisciplinary Research, Development and Innovation of the University of Szeged, H-6720 Szeged, Hungary; 5 HUN-REN-SZTE Neuroscience Research Group, Hungarian Research Network, Danube Neuroscience Research Laboratory, University of Szeged (HUN-REN-SZTE), Tisza Lajos krt. 113, H-6725 Szeged, Hungary; 6 Department of Obstetrics and Gynecology, Semmelweis University, Üllői Út 78/A, H-1182 Budapest, Hungary; anicka.alexandra@semmelweis.hu

**Keywords:** oral microbiome, dsybiosis, periodontal disease, caries, oral candidiasis, oral cancer, kynurenine pathway

## Abstract

The oral microbiome, comprising bacteria, fungi, viruses, and protozoa, is essential for maintaining both oral and systemic health. This complex ecosystem includes over 700 bacterial species, such as *Streptococcus mutans*, which contributes to dental caries through acid production that demineralizes tooth enamel. Fungi like Candida and pathogens such as *Porphyromonas gingivalis* are also significant, as they can lead to periodontal diseases through inflammation and destruction of tooth-supporting structures. Dysbiosis, or microbial imbalance, is a key factor in the development of these oral diseases. Understanding the composition and functions of the oral microbiome is vital for creating targeted therapies for these conditions. Additionally, the kynurenine pathway, which processes the amino acid tryptophan, plays a crucial role in immune regulation, neuroprotection, and inflammation. Oral bacteria can metabolize tryptophan, influencing the production of kynurenine, kynurenic acid, and quinolinic acid, thereby affecting the kynurenine system. The balance of microbial species in the oral cavity can impact tryptophan levels and its metabolites. This narrative review aims to explore the relationship between the oral microbiome, oral diseases, and the kynurenine system in relation to certain systemic diseases.

## 1. The Human Microbiome: Focus on the Oral Microbiome

The human microbiome refers to the collection of all microorganisms, including bacteria, archaea, viruses, and fungi, that reside on and within the human body. These microorganisms inhabit various niches such as the skin, oral cavity, gastrointestinal tract, respiratory tract, and urogenital tract. The human microbiome plays a crucial role in maintaining health, influencing processes such as digestion, immune response, and protection against pathogens. The composition of the human microbiome is highly diverse and varies significantly between different body sites and among individuals [1]. This diversity is influenced by factors such as diet, genetics, age, environment, and lifestyle [2]. The establishment of the microbiome begins at birth, with initial colonization influenced by the mode of delivery (vaginal birth versus cesarean section) and maternal microbiota [3]. The microbiome undergoes significant changes during early childhood, stabilizing around the age of three [4]. However, it continues to evolve throughout life, with notable shifts occurring due to diet changes, illness, antibiotic use, and aging [5].

**Figure 1:**
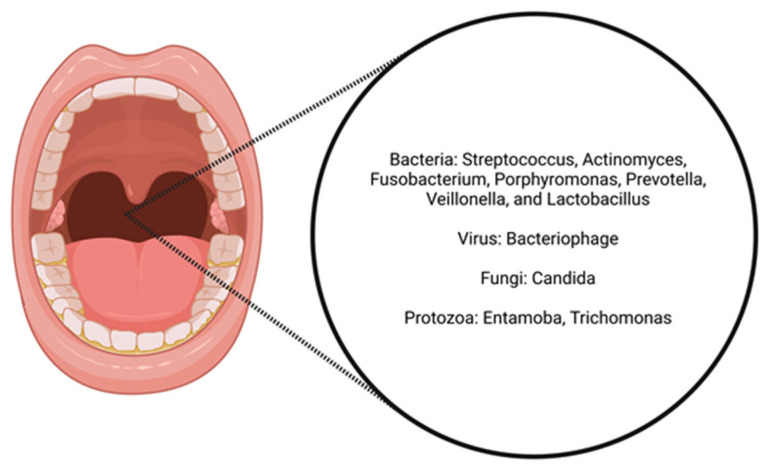
Key microbial components of the oral microbiome.

The oral cavity microbiome is a highly diverse and dynamic community of micro-organisms, including bacteria, viruses, fungi, and archaea, that inhabit various surfaces within the mouth, such as the teeth, gums, tongue, cheeks, and palate. The oral micro-biome plays a crucial role in maintaining oral health and overall systemic health, and it is composed of over 700 different microbial species, with bacterial phyla including *Firmicutes, Bacteroidetes*, *Proteobacteria*, *Actinobacteria*, *Fusobacteria*, and *Spirochaetes*. Key genera include *Streptococcus*, *Actinomyces*, *Fusobacterium*, *Porphy-romonas*, *Prevotella*, *Veillonella*, and *Lactobacillus*. In addition to bacteria, bacteriophages are typically present in the oral microbiome, *Candida* and other fungal species of the *Saccharomycetaceae* family are found within the fungal group [6], and *Entamoeba* and *Trichomonas* are found within the group of protozoa [7] (Figure 1).

The establishment of the oral microbiome begins at birth, with initial colonization influenced by the mode of delivery and the microbiome of the mother. As an individual grows, factors such as diet, oral hygiene, genetics, and environmental exposures further shape the oral microbiome [8]. The oral microbiome helps maintain oral homeostasis by preventing colonization by pathogenic microorganisms, contributing to the integrity of the mucosal barrier, and aiding in the digestion of certain foods [9]. In addition to this, the oral microbiome interacts with the host’s immune system [10], promoting the development and regulation of oral immune responses. It helps in the maturation of immune cells [11] and the production of antimicrobial peptides [12]. Oral microbes are involved in the metabolism of dietary sugars [13], which can lead to the production of acids that play a role in dental caries formation. They also contribute to the metabolism of nitrogenous compounds and the maintenance of the oral pH balance [14] and cardiovascular functions [15]. Oral dysbiosis refers to an imbalance in the microbial community of the mouth. Dysbiosis occurs when harmful microorganisms outnumber beneficial ones, leading to oral health issues. The pathological changes in the oral cavity’s microbiome remain largely underexplored, with most existing research primarily focusing on the overgrowth of *Candida albicans* in individuals with compromised immune systems or diabetes mellitus [16].

In contrast, viruses are typically found in the oral cavity only under pathological conditions, such as in cases of Acquired Immunodeficiency Syndrome (AIDS) [17] or infections caused by *Human Herpesvirus* and *Cytomegalovirus*. The bacteria that can be highlighted in relation to oral dysbiosis are: *Streptococcus mutans* [18], *Porphyromonas gingivalis* [19], *Fusobacterium nucleatum* [20], and *Actinomyces* spp. [21]. In addition to *Candida albicans*, *Candida glabrata* [22] also plays an important role in dysbiosis, particularly in individuals with chronic conditions.

Oral dysbiosis results from complex interactions between various microbial species and external factors. Understanding these roles helps in developing targeted therapies and preventive measures for oral diseases.

## 2. The Kynurenine Pathway

**Figure 2:**
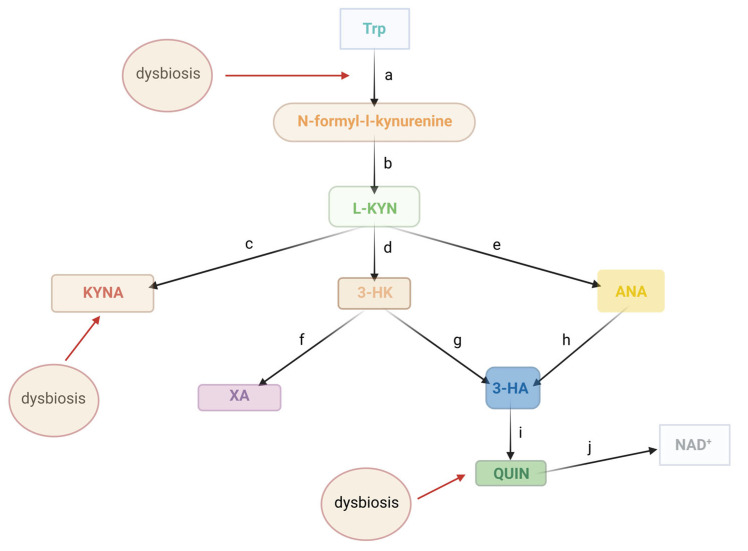
Main substances of the kynurenine system. This is a schematic summary figure of the KP and potential modulation points influenced by dysbiosis. A detailed description of the KP is available in these publications [32,33]. The abbreviations represent the individual components of the kynurenine system. Abbreviations: 3-HA—3-hydroxyanthranilic acid, 3-HK—3-hydroxykynurenine, ANA—anthranilic acid, KYNA—kynurenic acid, L-KYN—L-kynurenine, NAD+—nicotinamide adenine dinucleotide, QUIN—quinolinic acid, Trp—tryptophan, XA—xanthurenic acid. the black arrows indicate the steps of the kynurenine pathway, the red arrows show the potential influences of dysbiosis on the kynurenine pathway.

Tryptophan (Trp) is an essential amino acid vital for brain function as the precursor to serotonin (5-HT). Certain bacterial strains can boost Trp production in the body, which may help maintain regular gut motility [23]. However, in mammalian cells, over 90% of Trp is metabolized through the kynurenine pathway (KP) (Figure 2) rather than being converted to 5-HT. Among the key metabolites of the KP is kynurenic acid (KYNA), first identified by Justus von Liebig in 1853 in dog urine [24]. KYNA acts as an endogenous glutamate receptor antagonist with neuroprotective properties. It is synthesized from L-kynurenine (L-KYN) by kynurenine aminotransferases (KATs) (Step c, Figure 2). L-KYN itself is produced from L-Trp via the action of iron-dependent enzymes—indolamine 2,3-dioxygenase 1 and 2 (IDO1 and IDO2) and tryptophan 2,3-dioxygenase (TDO) (Step a)—through an intermediate, N-formylkynurenine (Step b). Beyond KYNA, L-KYN can be converted into anthranilic acid (ANA) via kynureninase (3-HAO) (Step e) or into 3-hydroxykynurenine (3-HK) via kynurenine 3-monooxygenase (KMO) (Step d), a mitochondrial protein in eukaryotic cells. ANA can further transform into 3-hydroxyanthranilic acid (3-HAA) through the action of 3-hydroxyanthranilic acid hydroxylase (Step h), while 3-HK can also convert into 3-HAA (Step g) or xanthurenic acid (Step f). Additionally, 3-HAA can be further metabolized into quinolinic acid (QUIN) by 3-hydroxyanthranilic acid 3,4-dioxygenase. QUIN (Step i) is then converted to nicotinamide adenine dinucleotide (NAD+) (Step j), which plays a crucial role in mitochondrial energy management and redox reactions. Kynurenines are also involved in regulating immune responses, sometimes acting as immunosuppressive agents and other timtes contributing to inflammation depending on the specific metabolite and context. For example, L-KYN itself can modulate immune cell activity and has been implicated in the regulation of T-cell responses [25]. Research has highlighted the critical roles of KP enzymes and metabolites in the pathomechanisms of migraine [26,27], Parkinson’s disease [28], mitochondrial dysfunction [29], schizophrenia [30], and, more recently, in the context of oral diseases [31]. The “Janus face” nature is particularly relevant in neurodegenerative diseases, psychiatric disorders, and immune dysregulation, where the balance of these metabolites may shift, contributing to disease progression or protection [29].

## 3. The Oral Microbiome and the Kynurenine Pathway: Key Players in the Pathomechanism of Oral Disorders

### 3.1. Kynurenines in the Context of Periodontal Diseases

Periodontal diseases, including gingivitis and periodontitis, are inflammatory conditions affecting the gums and supporting structures of the teeth. They are associated with dysbiosis in the subgingival microbiome, with increased proportions of pathogenic bacteria like *Porphyromonas gingivalis*, *Tannerella forsythia*, and *Treponema denticola* [34]. Gingivitis is a common form of gum disease characterized by inflammation of the gingiva, the part of the gum around the base of the teeth. It is a mild, early stage of periodontal disease and is typically reversible with proper oral hygiene. The primary cause of gingivitis is the accumulation of plaque, a sticky film composed of bacteria, food particles, and saliva. If not removed, plaque can harden into tartar, leading to further irritation and inflammation. If left untreated, gingivitis can progress to periodontitis, a more severe form of gum disease that can lead to tooth loss and other health issues. Periodontitis involves the destruction of the tissues that support the teeth, including the bone. Gingivitis and oral dysbiosis are closely related. Dysbiosis can lead to the overgrowth of pathogenic bacteria that cause gum inflammation, triggering gingivitis. Conversely, untreated gingivitis can further disturb the balance of the oral microbiome, perpetuating a cycle of dysbiosis and gum disease. Although taxa such as *Neisseria* spp. and *Streptococcus* spp., which consume oxygen, are also present in higher numbers during gingivitis, the condition is primarily associated with an enrichment of anaerobic species [35]. Key microorganisms involved include species from the *Streptococcus* [36] to *Actinomyces* [37] and *Fusobacterium* [38]. Periodontitis is a more severe form of periodontal disease that leads to irreversible damage to the periodontal ligament and alveolar bone. Key pathogens associated with periodontitis are collectively referred to as the “red complex,” which includes *Porphyromonas gingivalis*, *Tannerella forsythia*, and *Treponema denticola* [39]. In the initial stages of periodontitis, resident cells of the innate immune system, along with phagocytic cells, complement proteins, and neuropeptides, trigger an acute inflammatory response in reaction to supragingival and subgingival plaque. Pro-inflammatory cytokines like tumor necrosis factor (TNF), interleukin-1 (IL-1), and interleukin-6 (IL-6) are central to this process, driving the migration of cells to the infection sites. These cytokine clusters (IL-1, IL-6, and TNF families) contribute to periodontal damage by inducing the expression of the RANKL receptor, which is critical for osteoclast maturation and activation, ultimately leading to alveolar bone loss [40,41].

The interaction between the oral microbiome and the kynurenine pathway (KP) can influence oral and systemic health through several mechanisms. The oral microbiome can influence local and systemic immune responses. Microbial products and metabolites may interact with host cells to modulate the KP [42], affecting immune cell function and inflammation. Periodontal pathogens such as *Porphyromonas gingivalis*, *Tannerella forsythia*, and *Treponema denticola* can induce inflammation and disrupt immune homeostasis [43], demonstrating the role of microorganisms in inflammatory and various immunological processes. It should be emphasized that inflammatory cytokines, such as interferon-gamma (IFN-γ) and TNF-α, which are elevated in periodontal disease [44], can activate IDO [45], the rate-limiting enzyme of the KP, supporting the link between the oral microbiome and the KP. This process can lead to increased Trp catabolism and elevated levels of L-KYN and its metabolites. In addition, the expression of IDO was higher in periodontitis lesions compared to healthy gingiva [46]. It is crucial to understand that increased KP activity has been associated with cardiovascular diseases [47], diabetes [48], and neuroinflammation [49]. QUIN has pro-inflammatory properties. Chronic inflammation plays a central role in periodontal disease, and elevated levels of inflammatory mediators in the oral cavity could exacerbate gum disease and other oral inflammatory conditions.

**Figure 3:**
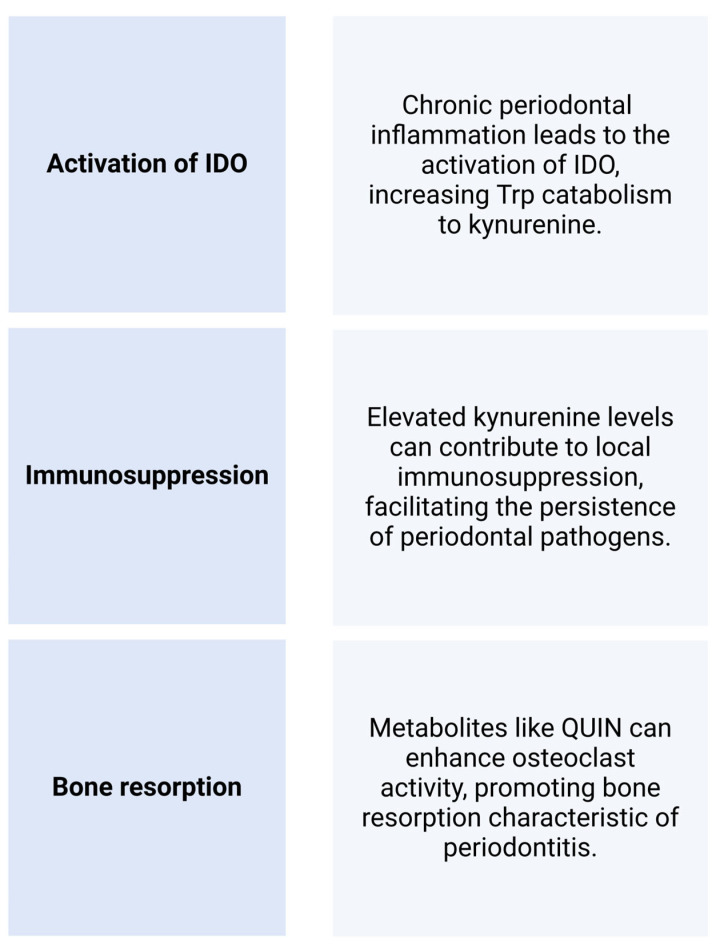
Periodontal diseases are strongly associated with the kynurenine pathway [31,50,51]. Abbreviations: IDO—indolamine 2,3-dioxygenase, QUIN—quinolinic acid, Trp—tryptophan.

Periodontal disease, through its inflammatory burden, may contribute to the activation of the KP and exacerbate these conditions. To conclude, the oral microbiome has been linked to neuroinflammatory conditions through mechanisms involving systemic inflammation, bone resorption, and the KP (Figure 3).

### 3.2. The Possible Role of Kynurenines in Caries

Dental caries, commonly known as tooth decay, is one of the most prevalent oral diseases. Dental plaque is a biofilm formed by the accumulation of bacteria on the tooth surface, and this biofilm is a key factor in the development of caries. The formation of dental plaque involves three critical steps [52]: First, immediately after tooth cleaning, salivary molecules adsorb to the enamel, creating a complex coating of glycoproteins, acidic proline-rich proteins, mucins, bacterial cell debris, exoproducts, and sialic acid. Next, bacteria interact with this acquired pellicle through specific cell-to-surface interactions. Primary colonizers, primarily *Streptococcus* and *Actinomyces* species, establish the biofilm, which is influenced by factors such as osmolarity, carbon sources, and pH. In the final step, additional bacteria, including *Streptococcus mutans*, adhere to these primary colonizers through cell-to-cell interactions, leading to the formation of a biofilm on the tooth surface, commonly known as dental plaque. Key contributors to dental caries include *Streptococcus mutans* [52], a major cariogenic bacterium that thrives in acidic environments and produces lactic acid from fermentable carbohydrates, and *Lactobacillus* species [53], which play a significant role in the progression of advanced lesions. The following *Lactobacillus* species are most commonly associated with the development of caries in both children and adults: *Lactobacillus salivarius*, *Lactobacillus plantarum*, *Lactobacillus gasseri*, *Lactobacillus fermentum*, *Lactobacillus rhamnosus*, and *Lactobacillus casei/paracasei* [53].

While the direct link between the KP and dental caries is less established, the immune responses and inflammatory processes modulated by the oral microbiome can indirectly influence caries development. Bacterial acids produced by cariogenic bacteria like *Streptococcus mutans* lead to enamel demineralization [54]. Inflammation resulting from secondary infections or pulpitis may activate the KP, influencing local immune responses.

To summarize, the KP can suppress or modulate immune responses, which may affect inflammatory processes in oral tissues. Dental caries is primarily associated with bacterial infections, particularly *Streptococcus mutans* [52], and the host’s immune response plays a crucial role in controlling bacterial growth and preventing damage to tooth structures [52]. The anti-inflammatory effects of kynurenine metabolites could influence caries progression by altering local immune responses [25]. Inflammation is a key factor in the development of dental caries, as it leads to tissue damage and increases the risk of infection [54]. Certain KP metabolites, such as KYNA, exhibit anti-inflammatory properties that may reduce inflammation in oral tissues and slow the progression of caries [55]. Additionally, the KP contributes to oxidative stress, particularly through metabolites like QUIN. Oxidative stress is known to exacerbate caries by promoting tissue damage and creating a more favorable environment for bacterial growth [56]. The balance of kynurenine metabolites may influence oxidative stress levels in the oral cavity, thereby impacting the risk of caries.

### 3.3. Kynurenines in Oral Candidiasis

This fungal infection, caused mainly by *Candida albicans*, occurs when the balance of the oral microbiome is disrupted, often due to factors such as antibiotic use [57], immunosuppression [58], or diabetes [59]. *Candida* species can overgrow, leading to white plaques on the oral mucosa, which can be painful and may cause dysphagia (difficulty swallowing).

*Candida albicans*, the primary cause of oral candidiasis, can influence and be influenced by the KP. *Candida* infections can modulate Trp metabolism, affecting immune responses. A recent study found that patients with candidemia had significantly higher levels of IL-17A and L-KYN compared to those without candidemia [60]. The significant correlation between IL-17A and L-KYN levels with candidemia suggests their potential as biomarkers, and understanding the interaction between the oral microbiome, oral diseases, and the KP could guide therapeutic strategies. Furthermore, a recent study found that *Candida albicans* infection activates the aryl hydrocarbon receptor (AhR), which is responsive to KYNA. Additionally, treating oral epithelial cells with IFN-γ inhibits fungal endocytosis by inducing the production of kynurenines, leading to prolonged AhR activation [61].

### 3.4. Linking Kynurenines to Oral Cancer

The oral microbiome is implicated in the development and progression of oral cancers. Certain bacterial species are associated with an increased risk of oral squamous cell carcinoma (OSCC), possibly through mechanisms involving chronic inflammation and the production of carcinogenic metabolites. OSCC is the most common type of oral cancer, accounting for over 90% of cases [62]. Clinically, OSCC often presents as a red and white or purely red lesion with a slightly uneven surface and well-defined borders [63]. Early-stage lesions usually do not cause pain, but as they advance, they may induce discomfort by developing features such as ulceration, nodularity, and attachment to deeper tissues [64]. Ulceration is a frequent indicator of OSCC, marked by an uneven floor and edges [65]. The posterior lateral border of the tongue is the most frequent site, representing about 50% of cases [66]. OSCC primarily spreads to the ipsilateral lymph nodes in the neck through lymphatic channels, but it can also affect bilateral or contralateral lymph nodes [62]. *Fusobacterium nucleatum* [67] and *Porphyromonas gingivalis* [68] are particularly important bacteria associated with OSCC.

The connection between oral cancer and the KP is a subject of active and intense research. L-KYN is among the most powerful immunosuppressive metabolites, significantly contributing to the development of malignancies [69]. This has prompted researchers to investigate whether targeting the enzymes responsible for its synthesis might be an effective therapeutic approach for various cancers. So far, numerous preclinical and clinical studies have concentrated on this area, especially on inhibiting IDO activity [69,70], yielding noteworthy results. A study found a connection between oral cancers and the KP, showing that IFN-γ-induced IDO1 activation triggered the Trp-KYN-AhR signaling pathway, which enhanced stemness in OSCC cells and contributed to the tumor’s entry into immunological dormancy [71]. Moreover, serum response factor overexpression regulated the novel IDO1/Kyn-AhR signaling pathway, promoting OSCC cell migration and invasion by influencing the epithelial-to-mesenchymal transition [72]. Furthermore, inhibition of IDO by 1-methyl-tryptophan (1-MT, IDO inhibitor) or dinaciclib, which is recognized as an indirect inhibitor of this enzyme, showed a synergistic effect when co-administered with temozolomide in head and neck squamous cell carcinomas [73,74].

## 4. Impact of the Oral Microbiome on Systemic Disorders

**Figure 4:**
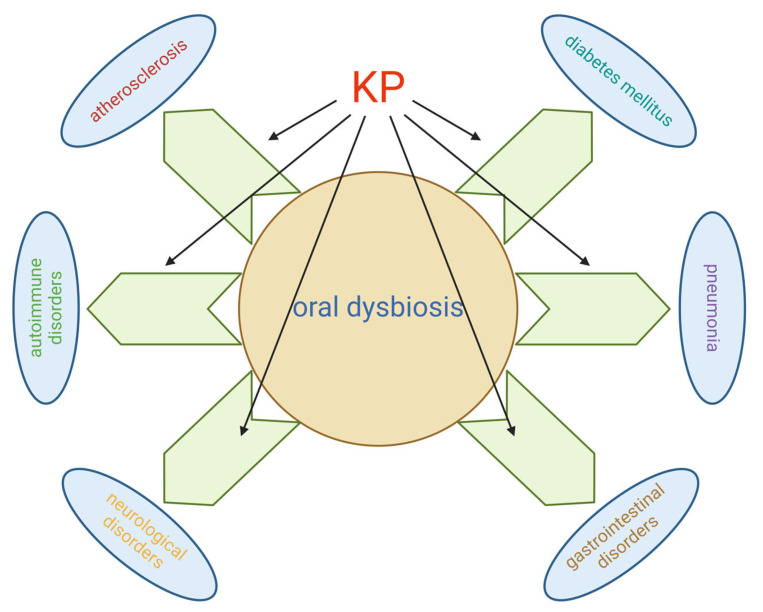
A comprehensive overview of the oral microbiome and kynurenine pathway, highlighting their contributions to the pathogenesis of various diseases.

The oral microbiome can influence systemic health through mechanisms such as bacteremia leading to endocarditis, systemic inflammation contributing to atherosclerosis, impaired glucose metabolism exacerbating diabetes, and contributing to conditions like rheumatoid arthritis, pneumonia, and gastrointestinal and neurological disorders (Figure 4).

### 4.1. How the Oral Microbiome Relates to Atherosclerosis

The relationship between the oral microbiome and atherosclerosis is an area of growing interest in research [75]. Atherosclerosis, a condition characterized by the buildup of plaques in the arterial walls, can lead to serious cardiovascular diseases, including heart attacks and strokes [76]. The connection between the oral microbiome and atherosclerosis involves several key mechanisms. Inflammatory conditions like periodontitis can lead to chronic inflammation [77], with inflammatory mediators from oral infections entering the bloodstream and contributing to systemic inflammation—a significant factor in atherosclerosis development. Oral pathogens also trigger the release of pro-inflammatory cytokines [78], promoting the formation of atherosclerotic plaques. Notably, certain bacteria, such as *Porphyromonas gingivalis* [79,80] and *Streptococcus mutans* [81], have been found in atherosclerotic plaques, suggesting a direct role in plaque development. Additionally, some oral bacteria may influence lipid metabolism, leading to increased low-density lipoprotein (LDL) cholesterol in rats [82], a major atherosclerosis risk factor. Bacterial enzymes might further modify lipoproteins, enhancing their contribution to plaque formation [83]. It is important to mention that some oral bacteria, such as *Porphyromonas gingivalis*, possess antigens that are similar to human proteins [84]. This molecular mimicry can lead to an autoimmune response where the body’s immune system attacks its own vascular tissues, contributing to endothelial damage and atherosclerosis [85]. The relationship between the oral microbiome and atherosclerosis is complex and involves multiple mechanisms, including systemic inflammation, molecular mimicry, and direct bacterial invasion of vascular tissues.

Elevated kynurenine levels have been associated with increased risks of coronary artery disease (CAD) and the progression of atherosclerosis [47]. This is due in part to the pathway’s influence on immune cells, such as macrophages, and the promotion of foam cell formation, which is critical to plaque development in arteries. Additionally, the pathway’s enzymes, like IDO1, are often upregulated in inflammatory conditions, contributing to endothelial dysfunction and the formation of atheromatous plaques [86]. Research also suggests that kynurenine metabolites can induce oxidative stress and inflammation [29], further exacerbating vascular disease.

### 4.2. The Impact of the Oral Microbiome on Type 2 Diabetes Mellitus and Vice Versa

The relationship between the oral microbiome and type 2 diabetes mellitus (DM 2) is bidirectional, with each influencing the other. One of the primary mechanisms by which the oral microbiome affects DM 2 is through periodontal disease [87]. Chronic inflammation associated with periodontal disease can elevate systemic inflammation, thereby contributing to insulin resistance [88], a key feature of DM 2. Patients with DM 2 frequently exhibit an altered oral microbiome, characterized by an overgrowth of pathogenic bacteria and a reduction in beneficial species [89]. This dysbiosis can exacerbate periodontal disease, creating a vicious cycle that further destabilizes glycemic control. Elevated blood glucose levels in diabetics contribute to the formation of advanced glycation end products (AGEs) [90], which accumulate in tissues, including the gingiva [91]. Moreover, AGEs exacerbate inflammation [92] and periodontal disease [93], further impairing blood glucose regulation. Numerous studies have demonstrated that non-surgical periodontal therapy, such as scaling and root planing, can significantly reduce glycated hemoglobin (HbA1c) levels in patients with DM 2 [94,95]. The observed improvement in glycemic control is largely attributed to the reduction in systemic inflammation following periodontal treatment. On the other hand, certain probiotic bacteria have shown promise in modulating inflammation [96,97]. Prominent among the commonly utilized probiotic strains are various species of *Lactobacillus* and *Bifidobacterium*, including *L. acidophilus*, *L. casei*, *L. gasseri*, *L. johnsonii*, *L. rhamnosus*, *L. reuteri*, *B. bifidum*, *B. infantis*, and *B. longum*. Notably, multiple studies have underscored the critical role of *L. gasseri* and *L. fermentum* in maintaining oral health by effectively inhibiting key periodontal pathogens, such as *Porphyromonas gingivalis*, *Prevotella intermedia*, and *Aggregatibacter actinomycetemcomitans* [98]. These probiotics may help restore microbial balance, thereby reducing the severity of periodontal disease and improving glycemic control. Additionally, combining probiotics with prebiotics (compounds that promote the growth of beneficial bacteria) is being explored as a strategy to enhance oral health and metabolic outcomes in diabetic patients [97]. The idea of transplanting a healthy oral microbiome to restore balance in individuals with severe dysbiosis is being explored as a potential treatment for both periodontal disease and diabetes [99]. This approach employs a technique similar to fecal microbiome transplantation (FMT), which involves transferring oral microbiota from a healthy donor to a patient suffering from different disorders.

The KP has gained attention due to its potential role in the pathophysiology of metabolic disorders, including DM2. Research suggests that chronic inflammation, commonly seen in DM2, can activate the KP [48], leading to an imbalance in kynurenine metabolites that may exacerbate metabolic dysfunction and insulin resistance. Inflammation in DM2 increases the expression of IDO [100,101], leading to higher kynurenine production. Certain metabolites, like QUIN, are linked to inflammation and oxidative stress, which can worsen insulin resistance [102]. In summary, the KP plays a role in modulating immune and metabolic processes that can influence the progression of DM2. Elevated levels of certain kynurenine pathway metabolites are linked to increased inflammation and insulin resistance, making it a potential area of therapeutic exploration in diabetes management.

### 4.3. From Mouth to Mind: The Role of Oral Microbes in Neurological Disorders

The link between the oral microbiome and neurological disorders is primarily based on the idea that imbalances in the oral microbiome can influence brain health through various mechanisms. Oral infections, particularly periodontal disease, can lead to chronic inflammation, as discussed earlier. This inflammation releases pro-inflammatory cytokines into the bloodstream, which can cross the blood–brain barrier and contribute to neuroinflammation [103]. Neuroinflammation, resulting from an innate immune response in the brain, can occur in two forms—an acute form with temporary expression of inflammatory mediators, and a chronic form where the resolution phase is significantly prolonged. This process is a key factor in many neurological disorders, including Alzheimer’s disease, Parkinson’s disease (PD), and multiple sclerosis (MS).

Alzheimer’s disease is a progressive neurodegenerative disorder characterized by a gradual decline in cognitive functions, including memory, thinking, and reasoning. It is the most common form of dementia and typically affects older adults [104]. Data from the literature strengthens the connection with the oral microbiome; certain oral pathogens, such as *Porphyromonas gingivalis*, have been detected in the brains of patients with Alzheimer’s disease [105]. This bacterium may enter the brain either through the bloodstream or by traveling along nerves like the trigeminal nerve, which connects the oral cavity to the brain. Once in the brain, these pathogens can directly contribute to neurodegeneration, such as the formation of amyloid plaques in Alzheimer’s disease [106]. Taken together, proinflammatory mediators generated by inflamed gingiva can spread from periodontal pockets to the brain through the bloodstream or directly via the trigeminal nerve, while dysbiotic oral bacteria may indirectly disrupt central nervous system (CNS) homeostasis.

Oral pathogens may produce molecules that closely resemble the body’s own proteins. This mimicry can potentially trigger an autoimmune response, where the immune system mistakenly attacks the body’s own tissues, including those in the nervous system. This mechanism is suspected to play a role in conditions like MS [107]. MS is a chronic, autoimmune demyelinating disorder of the CNS characterized by the progressive destruction of myelin sheaths surrounding neuronal axons. This disruption of myelin impairs nerve conduction and leads to a wide range of neurological symptoms. The etiology of MS is multifactorial, involving genetic predisposition, environmental triggers, and immunological dysfunction. Studies have found that individuals with MS have differences in their oral microbiome compared to healthy controls [108,109]. These differences could involve variations in the abundance and diversity of bacterial species. Systemic inflammation and immune system alterations linked to dysbiosis can, naturally, affect both the onset of MS and its progression [110].

PD is a progressive neurodegenerative disorder characterized primarily by the degeneration of dopaminergic neurons in the substantia nigra, a key structure in the basal ganglia. This neurodegeneration leads to a deficiency of dopamine, a neurotransmitter crucial for coordinating movement. Oral dysbiosis has been linked to an increased risk of Parkinson’s disease, potentially through mechanisms involving systemic inflammation [111] and the direct spread of oral pathogens affecting dopaminergic neurons. In a particularly striking study, researchers identified a distinct microbiota profile in the saliva and subgingival dental plaque of PD patients compared to healthy controls [112]. PD patients showed reduced alpha-diversity in both plaque and saliva samples. Additionally, while microbial profiles in saliva were associated with cognitive function, salivary flow rate, and indicators of an increased risk of periodontal disease, the microbiota in dental plaque correlated with the severity of motor symptoms in PD [112].

While the connection between the oral microbiome and neurological disorders is promising, more research is needed to fully understand the mechanisms involved. It is still unclear whether oral dysbiosis directly causes neurological disorders or is simply associated with them. In addition to this, the oral microbiome is highly individual, influenced by factors like diet, genetics, and environment, making it challenging to generalize findings.

The KP plays a significant role in various neurological conditions, which have been summarized in numerous reviews [113,114,115,116,117]. The various neuroactive metabolites of the kynurenine pathway (KP) exert opposing effects on neurons. Some metabolites, such as picolinic acid, KYNA, and the cofactor nicotinamide adenine dinucleotide, are neuroprotective, while others, like 3-hydroxykynurenine and QUIN, are neurotoxic. Both alterations in metabolite levels and disruptions in their ratios, particularly the QIUN-to-KYNA ratio, have been implicated in numerous diseases. Moreover, enzymes involved in the KP play critical roles in modulating the immune system, supporting neuronal energy maintenance, and influencing redox processes and inflammatory cascades, highlighting a complex and interconnected biological system. The oral microbiome can influence the KP in the body through the mechanisms previously described.

### 4.4. The Relationship Between the Oral Microbiome and Pneumonia

Emerging research suggests that an imbalance in the oral microbiome can contribute to respiratory infections, including pneumonia. Pneumonia, an inflammatory condition of the lungs, is often caused by pathogens like bacteria, viruses, or fungi. The concept of an “oral–lung axis” is gaining attention. Research suggests that the microbiomes of the mouth and lungs are interconnected and can influence each other. Chronic periodontal disease has been associated with increased markers of inflammation in the respiratory tract, potentially contributing to a heightened risk of respiratory diseases, including pneumonia and chronic obstructive pulmonary disease (COPD) [118]. Studies have identified specific oral bacteria in the lungs of patients with pneumonia. *Streptococcus pneumoniae*, *Porphyromonas gingivalis*, and *Fusobacterium nucleatum*, which are common in oral biofilms, are often found in cases of bacterial pneumonia [119]. Aspiration of oral bacteria into the respiratory tract is a significant route for pneumonia development, especially in older adults and hospitalized patients. Poor oral health, especially in individuals with periodontal disease, increases the bacterial load in saliva, raising the risk of bacterial migration to the lungs. Research indicates that patients on mechanical ventilation who receive regular oral care experience a lower incidence of ventilator-associated pneumonia [120]. Maintaining a healthy oral microbiome through good oral hygiene practices may, therefore, play a role in reducing the risk of respiratory infections like pneumonia.

KP metabolites are known to influence lung immunity [121]. This suggests that if the oral microbiome shifts to a pro-inflammatory state, stimulating the KP may help moderate local inflammation but could also dampen the immune response in the lungs, increasing susceptibility to infections such as pneumonia. Kynurenine-to-Trp ratios have been proposed as biomarkers of immune activation and inflammation, and studies have shown that elevated kynurenine levels are associated with worse outcomes in respiratory infections [121]. Monitoring KP activity could provide insights into pneumonia risk, especially in people with chronic inflammatory conditions or oral dysbiosis.

### 4.5. The Interplay Between Gastrointestinal Conditions and the Oral Microbiome

Evidence increasingly suggests that the oral microbiome is closely linked to gastrointestinal (GI) disorders. Bacteria from the mouth can travel to the GI tract via saliva or swallowing, with species like *Porphyromonas gingivalis* found in the gut, where they disrupt the microbial balance and contribute to inflammatory GI conditions, such as inflammatory bowel disease (IBD) [122]. Once settled in the gut, these bacteria may release toxins or interact with the immune system [123], triggering inflammation that affects gut health. Oral diseases like periodontitis can provoke an immune response that increases systemic inflammation. This systemic inflammation may, in turn, exacerbate GI disorders such as Crohn’s disease and ulcerative colitis [124]. Additionally, compounds from inflamed oral tissues can enter the bloodstream, affecting immune function and potentially influencing the onset or severity of GI inflammation [125]. Certain oral bacteria, such as *Fusobacterium nucleatum*, have been specifically linked to colorectal cancer and other GI conditions [126]. This bacterium is believed to promote inflammation and disrupt the gut mucosal barrier, potentially allowing harmful microbes or toxins to access and damage intestinal tissues. This connection suggests a complex “oral–gut axis”, where oral microbiome health could directly impact GI health and disease outcomes.

Some metabolites in the KP, such as QUIN, can impact gut–brain interactions and compromise the integrity of the intestinal barrier. When these metabolites are overproduced, they may weaken the mucosal barrier, allowing harmful substances to enter the bloodstream and triggering inflammation [127]. This weakened gut barrier, commonly referred to as “leaky gut”, is often seen in inflammatory bowel disease (IBD) and other GI disorders [128], where increased permeability permits immune-reactive substances to cause systemic inflammation. Elevated levels of specific kynurenine metabolites are frequently observed in inflammatory bowel disease (IBD) patients and are believed to worsen symptoms by amplifying inflammatory responses [129]. Trp metabolism, particularly through the KP, also influences the gut microbial community. Shifts in this pathway can promote dysbiosis, an imbalance in gut bacteria associated with conditions like IBD and irritable bowel syndrome (IBS) [130]. Furthermore, imbalances in Trp metabolism may reduce 5-HT availability in the gut, affecting gut motility and exacerbating GI disorder symptoms [131]. Together, these impacts on inflammation, microbiota composition, and gut barrier integrity underscore the kynurenine pathway’s role in GI health and disease.

## 5. Conclusions

**Figure 5:**
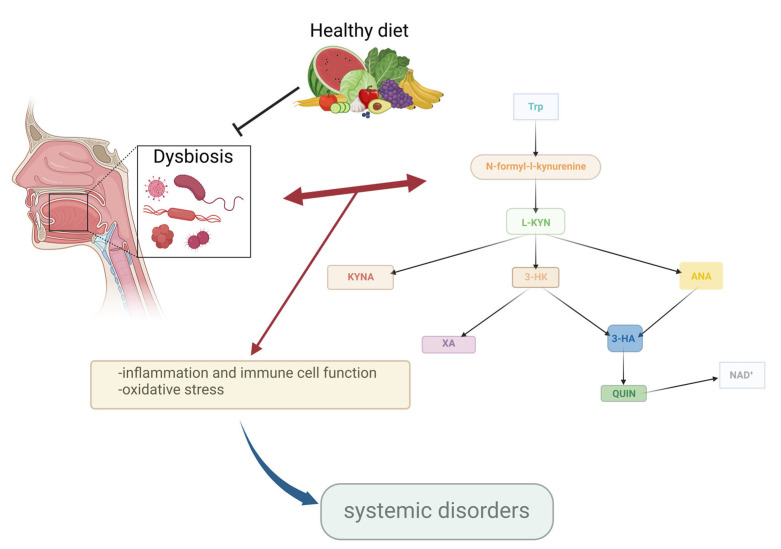
The bidirectional relationship between oral dysbiosis and the kynurenine system influences several processes that have been shown to play a role in the pathomechanism of various systemic disorders. Abbreviations: 3-HA—3-hydroxyanthranilic acid, 3-HK—3-hydroxykynurenine, ANA—anthranilic acid, KYNA—kynurenic acid, L-KYN—L-kynurenine, NAD+—nicotinamide adenine dinucleotide, QUIN—quinolinic acid, Trp—tryptophan, XA—xanthurenic acid.

Modulating the oral microbiome with probiotics and prebiotics is increasingly recognized as a crucial strategy for promoting a healthy microbial balance within the mouth, which, in turn, can have a significant impact on systemic health, particularly in the regulation of Trp metabolism. The oral microbiome plays a key role in the breakdown and utilization of nutrients, and its composition can influence metabolic pathways, including those involved in Trp metabolism. By fostering a favorable environment for beneficial bacteria, probiotics and prebiotics can help maintain oral health and potentially influence the KP, which is a major route of Trp degradation (Figure 5).

In addition to microbiome modulation, targeting inflammatory pathways and cytokines is another critical approach for influencing Trp metabolism. Chronic inflammation, often exacerbated by oral infections such as periodontal disease, can lead to the activation of IDO. This activation can shift Trp metabolism towards the production of kynurenines, some of which are neurotoxic and have been implicated in various systemic diseases, including neurodegenerative disorders. By reducing IDO activation through anti-inflammatory strategies, it is possible to modulate the KP and potentially mitigate its harmful effects.

Effective control of periodontal pathogens is essential not only for preventing local oral diseases but also for reducing systemic inflammation. By managing the oral microbial load and reducing periodontal inflammation, the systemic inflammatory burden can be decreased, which, in turn, may reduce the activation of the KP and its associated negative health impacts.

Diet also plays a significant role in maintaining oral and systemic health. Diets rich in anti-inflammatory nutrients, such as omega-3 fatty acids, antioxidants, and polyphenols, can support a healthy oral microbiome by reducing inflammation and promoting the growth of beneficial bacteria. Conversely, diets high in fermentable carbohydrates can lead to dysbiosis, or microbial imbalance, which can exacerbate inflammation and disrupt Trp metabolism. By adopting a diet that emphasizes anti-inflammatory foods and limits fermentable carbohydrates, individuals can support both oral health and the regulation of Trp metabolism, contributing to a more balanced and health-promoting metabolic profile.

In summary, we can conclude that the oral microbiome plays a significant role not only in oral diseases but also in the development of various systemic disorders. Modulating the KP could potentially have a positive impact on the oral microbiome, and the reverse may also hold true.

## Data Availability

Not applicable.
